# Analysis of the association between bladder carcinoma and arsenic concentration in soil and water in southeast Brazil

**DOI:** 10.1590/S1677-5538.IBJU.2017.0543

**Published:** 2018

**Authors:** Jonathan Doyun Cha, Danilo Budib Lourenço, Fernando Korkes

**Affiliations:** 1 Departamento de Urologia, Faculdade de Medicina do ABC, SP, Brasil; 2 Departamento de Urologia, Hospital Israelita Albert Einstein, São Paulo, SP, Brasil

**Keywords:** Arsenic, Urinary Bladder, Neoplasms

## Abstract

In approximately 50% of cases of bladder carcinoma, an associated predisposing factor can be established. The main factors are exposure to tobacco, arsenic (As) ore and aromatic compounds. Arsenic is a metalloid with a low average concentration in the earth's crust, and one of the most dangerous substances for human health. The present study aims to evaluate the incidence of hospitalization and mortality from bladder neoplasia and its possible association with As concentration in water and soil in two of the most critical regions of Brazil: the states of São Paulo and Minas Gerais.

We have investigated bladder cancer hospitalization and mortality in the states of Sao Paulo and Minas Gerais during 2010-2014. Water and soil samples were analyzed and As concentrations were established. Data were obtained through the Department of Informatics of the Brazilian Unified Health System. Correlation was made with water samples from São Paulo and with data on soil analysis from Minas Gerais.

The results revealed no direct association in the distinctive municipalities. Areas with high environmental As concentration had a low bladder cancer rate, while areas with normal as levels had similar cancer rates. The quantitative variables did not present a normal distribution (p < 0.05).

In conclusion, we did not observe a correlation between as concentration in water or soil and bladder cancer's hospitalization and mortality rates in the states of São Paulo and Minas Gerais.

## INTRODUCTION

Bladder cancer is the second most common neoplasm of the human urinary system, the ninth most common malignant neoplasm in the world, and the ninth most expensive neoplasm per patient from diagnosis to death in the United States (1–3). Along with lung neoplasm, bladder carcinoma was one of the first to be investigated for its epidemiology, and since 1954 studies have searched for carcinogenic substances (4). In approximately 50% of cases of bladder carcinoma, an associated predisposing factor can be established. The main factors are exposure to tobacco, *As* ore, aromatic compounds, hydrocarbons and chronic infections such as due to *Schistosoma haematobium* (3).

Arsenic is a metalloid with a low average concentration in the earth's crust (1.8 mm). It was classified by the agency for toxic substances and disease registry (ATSDR, 2007) (5) along with supplemental environmental projects of the United States (USEPA, 1998) as one of the most dangerous substances to human health. Its concentration in drinking water should not exceed 10-50 mcg / L and if surpass 50-100 mcg / L, there is evidence of adverse effects, making possible to detect its effects in epidemiological studies (6). According to the World Health Organization, human exposure to the ore occurs through the use of contaminated water, ingestion or inhalation of dust and inhalation of gases from various sources such as: pesticides, fertilizers, metal treatment (copper, lead and bronze), paint, glass, ammunition and galvanizing (7, 8).

Soil contamination by ores related to human activity has become the focus of many studies. Serious cases of *As* poisoning occurred in West Bengal, Bangladesh and, in Latin America, Mexico, Chile, and Argentina. These cases were generally caused by the consumption of contaminated groundwater extracted from aquifers in large arseniferous geological formations (9, 10). In the published inventories of environmental and human exposure to *As*, references to Brazil are scarce mainly due to the lack of research on the subject in the country.

In Brazil, there are three areas considered critical for the risks of exposure to As: (1) *‘Quadrilátero Ferrífero’,* in Minas Gerais, where a large amount of *As* was released as a result of secular gold mining; (2) Ribeira Valley, in Paraná and São Paulo, due to mining activity; (3) Santana, Amapá, where *As* was associated with manganese ore mining over the last 50 years. Specifically, in the states of Minas Gerais and São Paulo, there are studies evaluating the concentration of *As* in soil and water (11). The present study aims to evaluate the incidence of hospitalization and mortality from bladder neoplasia and its possible association with as concentration in water and soil in two of the most critical regions of Brazil: the states of São Paulo and Minas Gerais.

## MATERIALS AND METHODS

Between 2010 and 2014, all cases of bladder cancer have been evaluated through the registration of surgical procedures and mortality rates due to bladder carcinoma, in the states of São Paulo and Minas Gerais - Brazil. Data were obtained through the Department of Informatics of the Brazilian Unified Health System. In the state of São Paulo, water samples were analyzed and as concentrations were established through data provided *by Companhia Ambiental do Estado de São Paulo* (CETESB). Data on soil analysis from the state of Minas Gerais were collected and analyzed by the State Environmental Foundation (FEAM) under the *Solos de Minas* Program, in partnership with the Federal Universities of Viçosa, Lavras and *Ouro Preto and the Fundação Centro Tecnológico de Minas Gerais* (CETEC), through previously described methodology (12, 13). The municipalities with the highest concentration of *As* in the soil were compared to areas with low concentration. In the state of São Paulo, water was collected and analyzed by CETESB - São Paulo, from seven aquifers and 19 water management units. The municipalities with the highest *As* rates in its samples were compared to those with normal ranges.

### Analysis of soil and water samples

A total of 499 soil samples from 299 municipalities in the state of Minas Gerais were collected and analyzed. The municipalities with the highest concentrations of As in the soil were Mariana, Ouro Preto, Rio Acima, Caeté, Nova Lima, Santa Bárbara, Sabará and Itabirito, all above 8.6 mg kg ^-1^. These values are much higher than the acceptable upper limit of 1.0 mg kg-1 in soils not contaminated by anthropogenic sources (9, 10, 12).

In the state of São Paulo, the municipality of Piedade presented the highest *As* rates in its samples (0.045 mg / L, maximum allowed value: 0.01 mg / L - Potability Standard of Ordinance 2914 / 11 of the Ministry of Health), having the municipalities of Biritiba Mirim, Cajati and Miracatu presented isolated abnormal samples (13).

We have than evaluated the association between bladder cancer and *As* contamination using the Spearman correlation test. The confidence level adopted in the analysis was 95%. Statistical analysis was performed using statistical software Stata version 11.0 - StataCorp 1996-2017.

## RESULTS

### Bladder cancer's hospitalization and mortality rates

In the state of Minas Gerais, hospitalization rate for bladder neoplasia was 5.9 per 100.000 inhabitants and ranged from 4.2 to 7.7 hospitalizations per 100.000 inhabitants between 2008 and 2015. There were 2.5 men for each woman. Seventy percent of patients had 60 – 79 years.

In the state of São Paulo, hospitalization rate for bladder neoplasia was 9.0 per 100.000 inhabitants and ranged from 6.0 to 11.0 hospitalizations per 100.000 inhabitants between 2008 and 2015. There were 2.6 men for each woman, and 68.8% of patients had 60 – 79 years.

The hospitalization rate for bladder cancer in São Paulo is above the national average (8.0 per 100.000), and in Minas Gerais is below.

**Figure 1:**
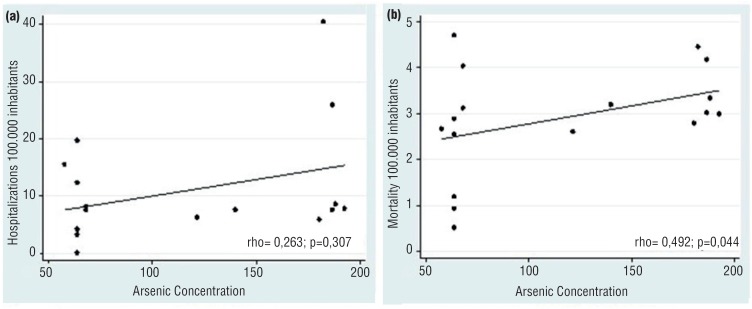
Association between arsenic contamination and hospitalization (a) and mortality (b) for bladder cancer in the state of São Paulo.

**Figure 2:**
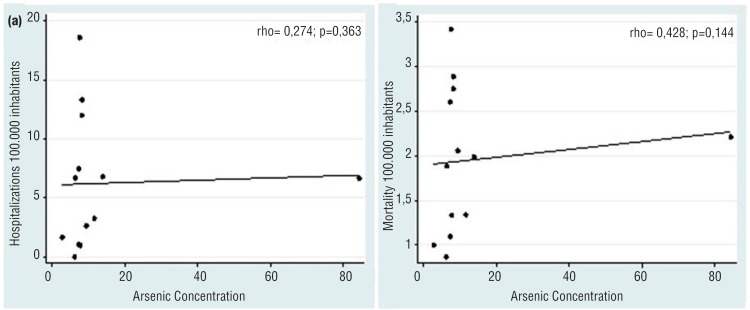
Association between arsenic contamination and hospitalization (a) and mortality (b) for bladder cancer in the state of Minas Gerais.

**Figure 3:**
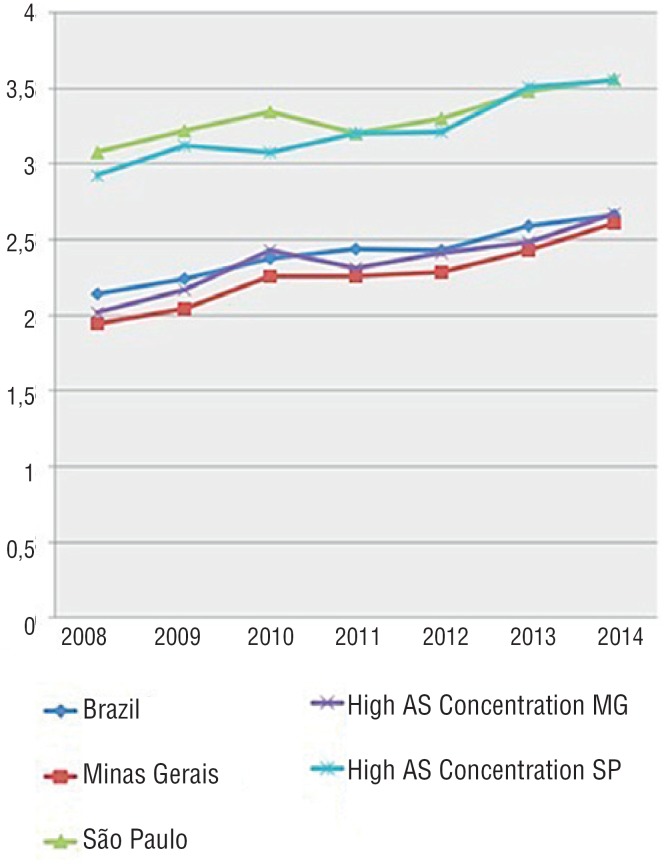
Mortality due to bladder neoplasia in Brazil, in the states of Minas Gerais and São Paulo, and their respective areas with the highest concentrations of arsenic.

**Figure 4:**
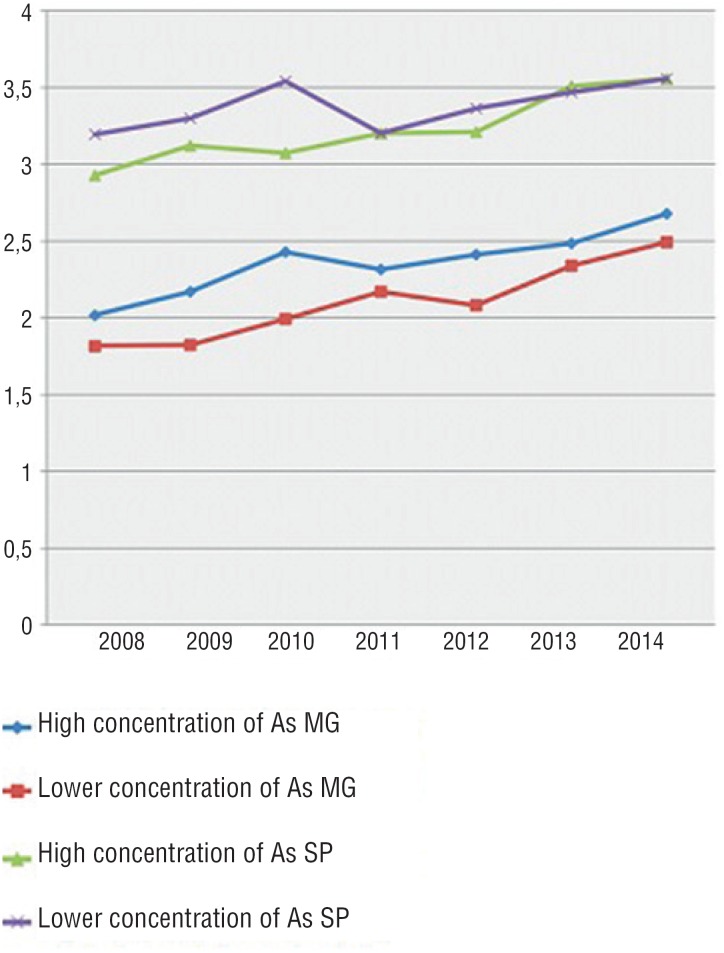
Mortality due to bladder neoplasia in regions with lower and higher concentration of arsenic in the states of São Paulo and Minas Gerais.

**Figure 5:**
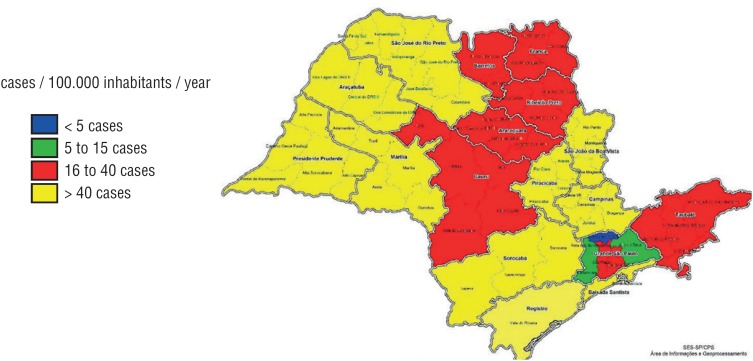
Map with the areas with high hospitalization rates (SP).

**Figure 6:**
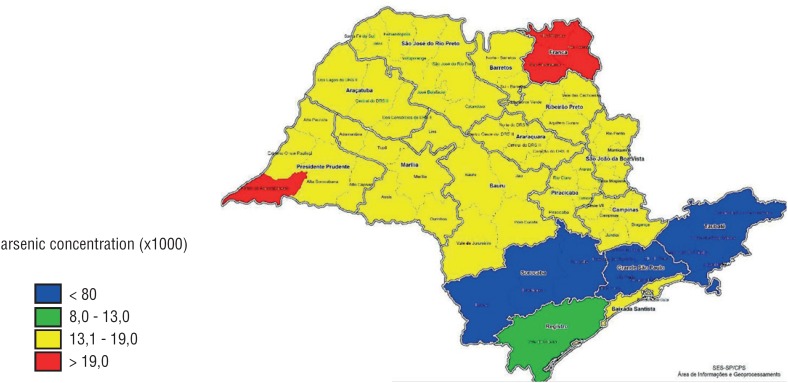
Map with the areas with high arsenic concentration (SP).

When bladder cancer cases were evaluated according to *As* concentrations in soil or water in the distinctive municipalities, we could not establish a direct association. Areas with a high environmental As concentration had a low bladder cancer rate, while areas with normal *As* levels had similar cancer rates (Figures 1–4). The quantitative variables did not present a normal distribution, using the Shapiro-Wilk test p < 0.05. We have also plotted a map with areas with high rates of hospitalization by bladder cancer (Figure-5) and *As* concentration (Figure-6) in São Paulo.

## DISCUSSION

Serious cases of *As* poisoning have been reported in distinctive countries (9, 10). These cases were generally caused by the consumption of contaminated groundwater extracted from aquifers in large arseniferous geological formations (9, 10). This greater exposure can translate into a higher concentration of *As* in the body. These high levels of As are associated with an increased risk of neoplasm. Argos et al. have demonstrated in a prospective cohort study in Bangladesh that there is an increase in the overall mortality rate in patients exposed to *As* in ingested water (14).

A Taiwanese study have compared bladder cancer mortality in general population and inhabitants of an area exposed to high concentrations of *As*, and established a direct and proportional relationship between the different urinary profiles of the ore and deaths by this type of neoplasia during 20 years of follow-up. Even after the source of exposure had been discontinued, overall mortality remained higher than in the general population (15). These risks can be potentialized by other carcinogens, such as those found in tobacco, the main epidemiological factor for bladder cancer. Pu ys et al. compared 313 cases with 177 controls and concluded that smoking interacts with the urinary *As* profile in the body, modifying the risk of urothelial carcinoma. The carcinogenic effect, however, was shown to be more prominent in non-smokers (16). Wu et al. also compared the risk of developing urothelial carcinoma according to smoking load and urinary as levels. Objects with high urinary levels of As (≥ 15.40 mcg / g creatinine) had a risk ratio of 3.2 when smoked less than 100 cigarettes during lifetime and 6.45 times greater when smoked more than this load, when compared to non-smokers and low urine levels (17). Interestingly, Mario Fernandez et al. reported that even 20 years after the normalization of the concentration of *As* in Chile's soil, the incidence of bladder cancer remained high (18).

In the published inventories of environmental and human exposure to As, references to Brazil are scarce mainly due to the lack of research on the subject in the country. In Brazil, there are three areas considered critical for the risks of *As* exposure: (1) *Quadrilátero Ferrífero,* in Minas Gerais, where a large amount of *As* was released as a result of secular gold mining; (2) Ribeira Valley, in Paraná and São Paulo, due to mining activity; (3) and Santana, Amapá, where as was associated with manganese ore mining over the last 50 years. Specifically, in the states of Minas Gerais and São Paulo, there are studies evaluating the concentration of as in soil and water (11). In critical areas, such as in the Ribeira Valley and Iron Quadrangle, less favored populations become more susceptible to exposure to these toxic substances. In the evaluated areas in this study, there is a combination of high natural rates of *As* associated with industrial and mining activity. Fortunately, low levels of human exposure of this toxic element are observed. Despite geochemical composition and risk activities, contaminated aquifers have not been described in Brazil until now, as in other regions of the world (10, 19). In a US National Cancer Institute survey, based on SEER (Surveillance, Epidemiology and End Results), mortality from bladder neoplasia was influenced by smoking, unemployment, days of altered physical health, days of air pollution, percentage of houses with well water, work in the ore industry and urban dwellings (p < 0.05) (20). In the present study, we did not find a direct relationship between higher concentrations of *As* ore in soil or water and higher rates of hospitalization or mortality from bladder neoplasia in the states of São Paulo and Minas Gerais, even when comparing the group with higher concentrations with the cities with lower concentration in each state (Figure-4). These findings may be justified by some factors.

The presence of *As* in deep water does not necessarily change the concentration of *As* in surface water and even in food. Even though some food contain *As* in their composition and the urinary profile is influenced by the ore methylation capacity of each individual, the consumption of fish, seafood or seaweed did not necessarily correlate with a change in the urinary *As* profile in residents of risk areas (21). Besides, the tropical climate favors the predominance of chemical weathering processes of the rocks, forming geochemical barriers that prevent the release of As to the water even in regions with high levels of this mineral. These processes justify the low concentrations of *As* in surface water, even when there are high concentrations of *As* in soils and sediments (11).

Our study has some limitations. Similar to other neoplasms, bladder cancer is possibly underreported as a cause of hospitalization or death, especially in poorer areas in developing countries, such as Brazil (22). Furthermore, the presence of incomplete demographic data and poor medical records may contribute to limit the understanding of the factors related to incidence, prevalence and mortality. It might also reflect the lack of access to health services in the region (22). Other important limitation is that, in order to be carcinogenic, a long term exposure to As high concentration is necessary. Along with these facts, there is a frequent interstate migration in Brazil. For these reasons, correlation between risk factors with bladder cancer is difficult to be established. How long must one person be exposed to As to suffer the effects of carcinogenicity is a question not yet answered. In mice experiments, urothelial changes happen within 12 weeks of exposure, but the same cannot be inferred to humans (23).

It seems however that bladder cancer related to as exposure is more aggressive and appears earlier in life (24). In the present study, we could not adjust the incidence for sex and age, and therefore could not evaluate any correlation. Although the present report has some limitations, to the best of our knowledge it is the only study that has evaluated the epidemiological correlation between as and bladder cancer in Brazil.

In conclusion, we did not observe a correlation between as concentration in water or soil and hospitalization and mortality rates due to bladder cancer in the states of São Paulo and Minas Gerais.
